# Remarks on Perfection in Plastic Surgery of the Face

**DOI:** 10.1155/2018/5835167

**Published:** 2018-01-16

**Authors:** Kazimierz Kobus, Katarzyna Kobus-Zaleśna

**Affiliations:** ^1^Hospital and Clinic of Plastic Surgery, Polanica-Zdrój, Poland; ^2^Department of Maxillofacial Orthopedics and Orthodontics, Wrocław Medical University, Wrocław, Poland

## Abstract

An expectation of perfect and close to ideal outcomes is attributed not only to aesthetic, but also to reconstructive surgery. Contrary to quite common belief and despite great potential, the real chances for achieving attractive appearance are limited to relatively simple cases with moderately abnormal morphology, sufficient homogenous material, and hardly conspicuous scars potential. Therefore, the expectations for fully satisfactory outcomes should be limited to jaw surgery, cosmetic rhinoplasty, otoplasty, and some rejuvenation procedures, provided the best and uneventful surgery is secured. On the basis of over 40 years of clinical practice (KK) and survey of about 30,000 photos presenting both early and long term outcomes, the authors present their own subjective opinion on the value and potential of plastic surgery with regard to aesthetic evaluation. The paper is illustrated by numerous examples of plastic and esthetic procedures.

## 1. Introduction

Striving for perfection and excellence in plastic surgery should be regarded as obvious and fully understandable. Good results of treatment are highly expected and even in extensive reconstructive procedures taken almost for granted. Quite often under influence of mass media plastic surgery is regarded as a synonym of ideal outcomes which due to many unfavourable factors are not so common however.

An important discrepancy between expectations and reality comes from human body defects and imperfections, not always optimal working conditions, deficient treatment, and many other not always predicted factors. Outcomes evaluation varies greatly, since the laymen opinions refer to normal appearance, while doctors appraisals are based mostly on preoperative abnormalities of anatomical structures and challenging replacement of specific original tissues.

## 2. Basic Conditionings

Apart from satisfactory health condition, the results of surgical treatment depend mostly on* morphology*, as well as* quantity and quality of material* needed for repair or reconstructions.

**Figure 1:**
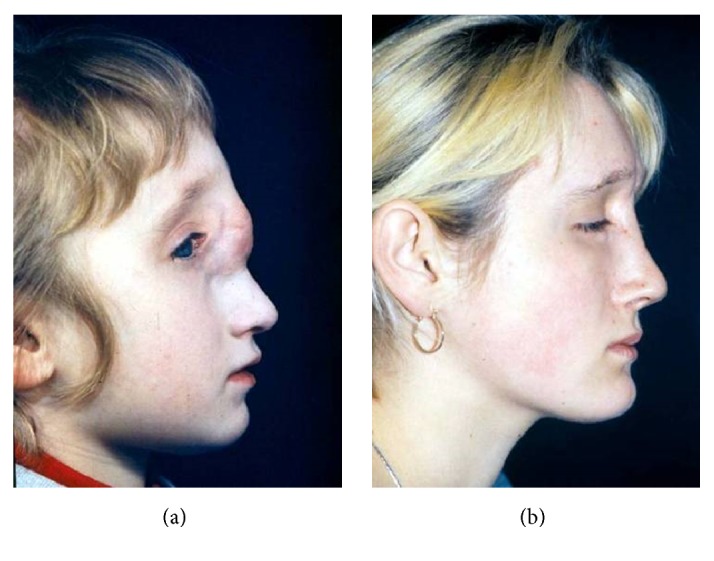
Hypertelorism and meningocele (a, b).

**Figure 2:**
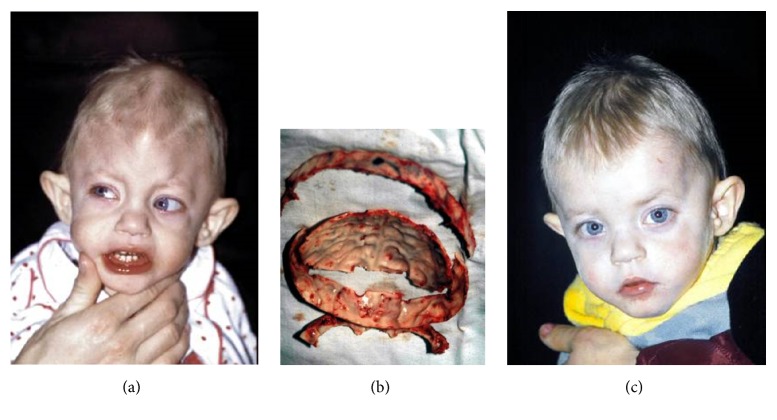
Craniostenosis (a, b, c).

Irrespective of origin, the repair of abnormalities, deformities, and misplacement of anatomic structures is relatively successful, provided there is no or only minimal deficit of tissues. The most difficult and challenging ones are craniofacial operations, but due to hidden scars even the correction of orbital dystopia, multiple craniostenoses, or severe jaw disorders can produce quite acceptable outcomes (Figures 1 and 2).

**Figure 3:**
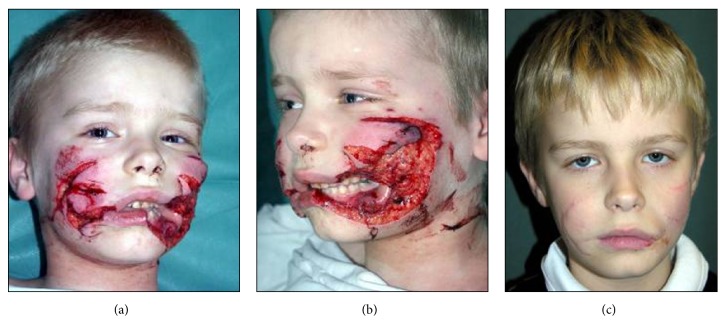
Facial injury with defect of tissues. ((a) and (b)) Before treatment. (c) Patient's appearance after cheek reconstruction with the use of tongue flap covered with skin graft, followed by skin graft excision and local skin plasty.

The management of patients with tissue defects, noted mostly in trauma and after resection of tumors, depends mostly on their size, localization, and facial nerve function. In patients with moderate deficiency of tissues the results of treatment are rather favourable, provided the local and possibly homogenous material can be used. In relatively extensive buccal defects the staged repair can be taken into consideration, which means the primary reconstruction with the use of unmatched filling material such as a distant or tongue flaps covered with skin grafts, followed by surrounding skin undermining and wound suturing, Figure 3.

Quite similar problem applies to many congenital malformations and especially to craniofacial and multiple clefts.

The management of incomplete clefts of the lip and palate or minor forms of hemicraniofacial microsomia (HCFM) is rather simple and spectacular, while, in severe malformations, perfect results of treatment occur rarely, due to important deficit of tissues and necessity of its replacement with substitute material [1, 2].

In HCFM classified as III grade, even if temporomandibular joint (TMJ), mandibular ramus, and soft tissue deficit are successfully reconstructed, the normal appearance can be achieved at rest only, because instead of proper levelling of dental occlusion some abnormalities can be detected while eating, speaking, and smiling.

**Figure 4:**
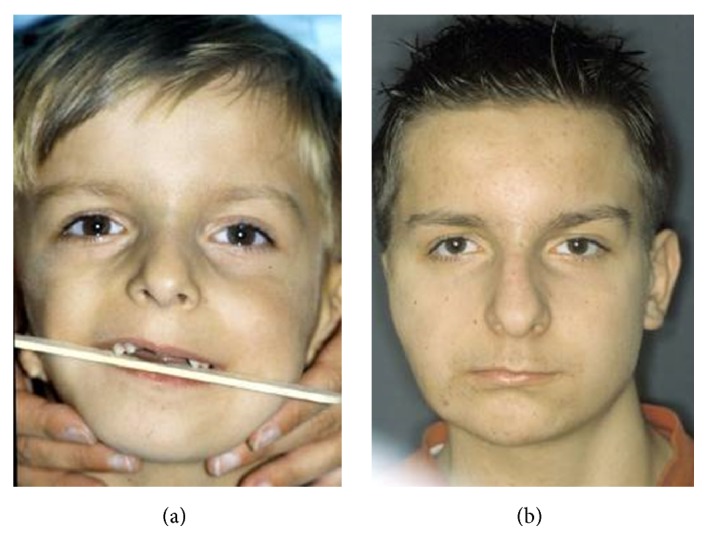
Severe HCFM before (a) and after (b) zygomatic arch, mandibular ramus, and auricular reconstruction.

Moreover an underdevelopment of the affected side of the face and low position of the auricular remnants make* restitutio ad integrum *futile and unrealistic, Figure 4.

**Figure 5:**
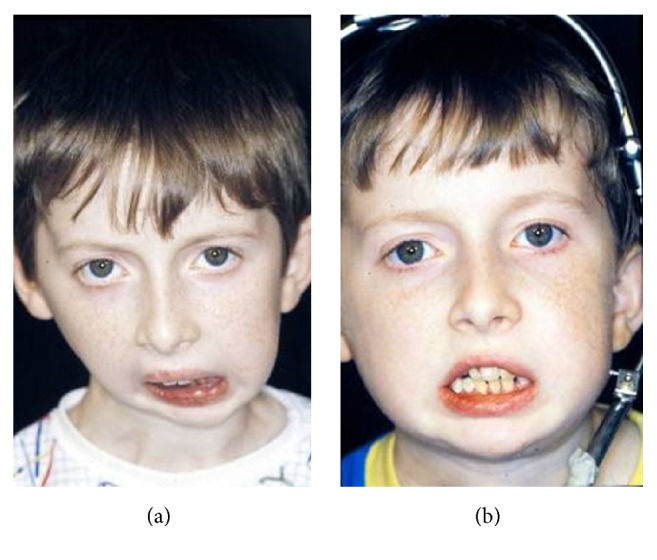
TMJ ankylosis repair. Mandibular ramus and condyle reconstruction with the use of costochondral graft.

The same applies to severe and long-lasting TMJ ankylosis, where despite successful repair some asymmetry is always noted, Figure 5.

It should be remembered that, in complete clefts, HCFM, TMJA, and other craniofacial malformations, the final outcomes depend also on the quality of orthodontic treatment [2]. It applies mostly to various forms of midface hypoplasia and asymmetries as well as protrusion of premaxilla in bilateral complete clefts.

Disregarding the less than optimal surgery, it should be remembered that, in many congenital abnormalities, too radical repair of young children can invite some problems with their* growth and development*. So, if there is a need for limited scope or staged procedures, the perfect outcomes can be expected only at the very end of treatment. Therefore, a complete cleft lip and palate repair evaluation is fully reliable after the growth period is accomplished.

The ideal replacement of specific facial tegument is often doomed to failure, and the only possibility for using homogenous material is local plasty or tissue expansion. In clinical practice the transposition of homogenous material is possible only in lid and lips. It should be remembered however that an expanded skin is rather not suitable for reconstruction of three-dimensional anatomic structures such as the nose, because its unavoidable postoperative shrinkage can impair the result of treatment.

**Figure 6:**
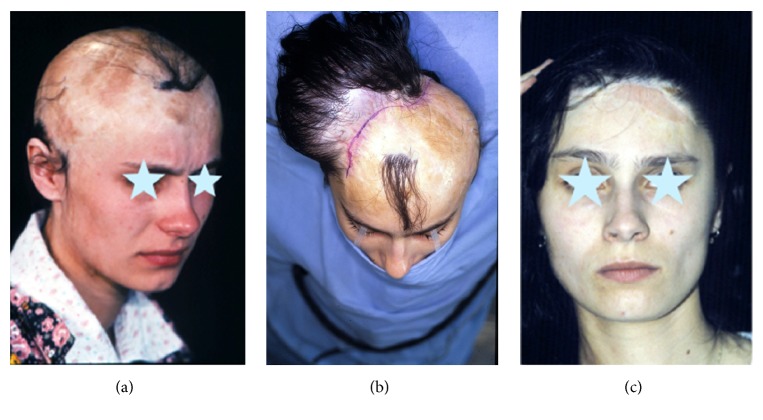
Posttraumatic loss of over 3/4 hair-bearing skin before (a), during expansion (b), and after four-stage treatment (c).

When concerning the hair-bearing skin expansion, a phenomenon beneficial but unclear to me is noted, which consists in better than expected hair density. So, the covering of even 3-4 times larger bald surfaces does not produce noticeable thin hair, Figure 6.

**Figure 7:**
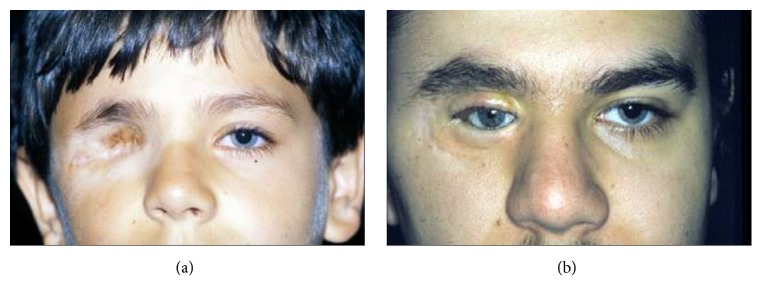
Eyelids reconstruction with prefabricated retroauricular flap according to author's method (KK). (a) Patient after orbit exenteration, before reconstruction. (b) Result of treatment.

Apart from local flaps and tissue expansion, in medium-size defects (up to 5 × 6 cm) the worthy solution is Washio method [3] or transposition of prefabricated retroauricular skin flaps [4, 5] which due to good color and consistency proves to be suitable mostly for reconstruction of the orbital and cheek regions, Figure 7.

In patients with significant deficit of tissues the limitation and uselessness of local plasty (“rob Peter to pay Paul”) extort the use of regional or even distant substitute material, whose color and texture preclude* restitutio ad integrum.* Therefore, the quality of the nose reconstructed according to the Indian method is almost always superior to this anatomic structure restored with the use of forearm, abdominal, or dorsalis pedis flaps.

The regional materials such as cervical or Bakamjian's flaps have admittedly quite acceptable properties, but their awkward transposition and donor site deformity limit their use and popularity, especially in women. The same can be said about bipedicled cervical flaps suitable for mandibular region reconstruction, because the secondary defects have to be covered with skin grafts.

**Figure 8:**
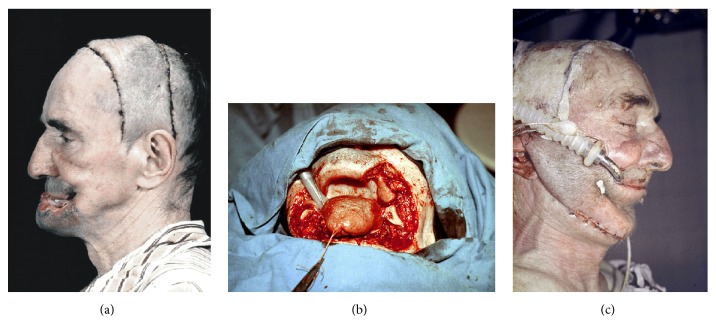
Lower lip and mandible cancer. Prefabricated Esser's flap (a). Intraoperative photo after tumor resection (b). Primary reconstruction of the lip, chin, and mandible (c).

In male patients bipedicled flaps raised from the scalp as visor Esser's flaps based on superficial temporal vessels are probably the better choice, especially in replacement of large and composed defects of the mandibular area, Figure 8.

Apart from identical twins, the almost equally matching material can be taken from other human beings. Growing popularity of facial allotransplantation (replacing like with like) is very spectacular indeed [6–8], but so far the problems related to lifelong immunosuppression and deficient motor innervation of reconstructed anatomical structures call for extreme moderation.

Moreover it should be remembered that on 31 transplantations performed between 2005 and 2015, four deaths occurred [7] while an expected beneficial application of chimeric cells is still uncertain [9]. It is a real dilemma, because the cosmetic results of facial transplantation are incomparably superior to traditional poor and tiresome reconstructions.

**Figure 9:**
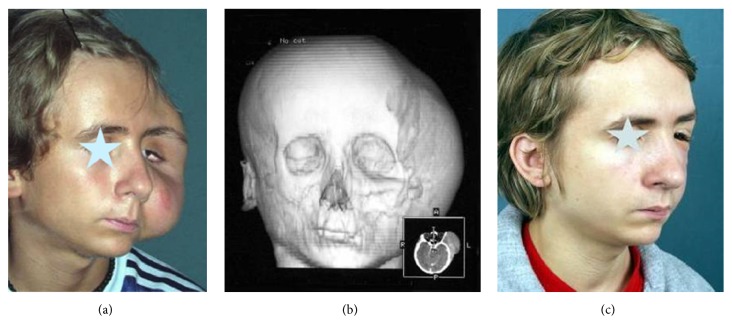
Patient with neurofibroma of the face (a), CT (b), and the case after tumor resection (c).

An* excess of tissues* precluding favourable outcomes applies mostly to cases characterized by concomitant hypertrophy of bony framework and facial tegument. In severe forms of facial neurofibromatosis an efficiency of surgical treatment is strongly limited. The most difficult and almost always doomed to failure one is eradication of tumors invading the orbits and anterior cranial fossa, so in such cases no one expects normal appearance. Much more can be done in patients with moderate involvement of facial tegument, although too radical excisions can result in mutilating facial nerve injury [10], Figure 9.

The same applies to removal of abnormal tissue in fibrous dysplasia of the orbits or to radical resection of the vascular tumors, due to the risk of devastating vision, massive haemorrhages, or facial nerve paralysis [10–13]. In life-threatening diseases the rationale for embolization followed by mutilating resections is evident, but in less severe ones, the palliative and less radical treatment is generally accepted. In some cases the wait-and-see attitude and staged repair prove to be reasonable and beneficial.

**Figure 10:**
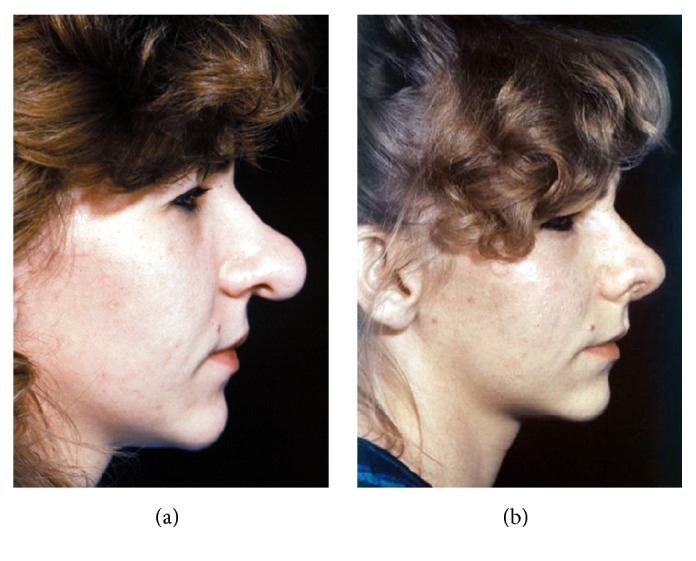
Big nose correction with the use of external incisions. (a, b).

In s*trict* cosmetic surgery the problem of big noses is not rare indeed, but a genuine hypertrophy in the form of Cyrano de Bergerac's noses is less common. Both in congenital as well as acquired forms, their modelling is rather efficient and despite postoperative scars at the borders of ala nasi, the outcomes are quite acceptable, however, Figure 10.

**Figure 11:**
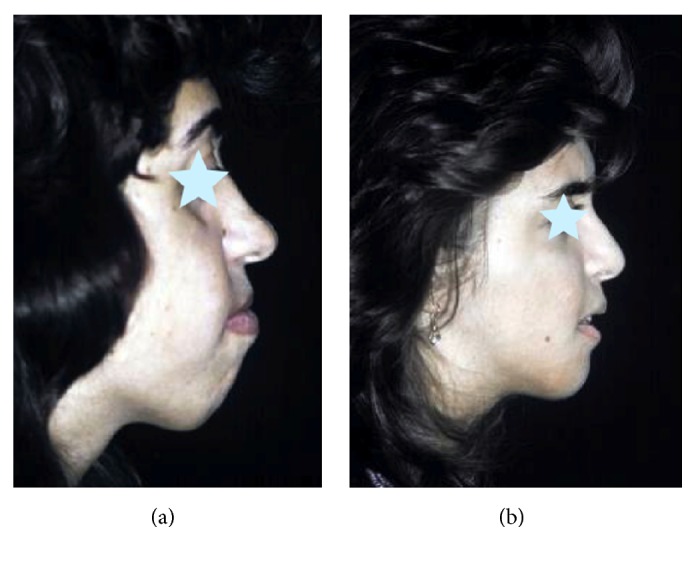
Facial deformity before (a) and after (b) bijaw osteotomy followed by rhinoplasty and chin advancement.

An impact of* scars* on surgical outcomes varies greatly. The least conspicuous marks are observed after ophthalmic, ENT, and maxillofacial operations, because an intraoral approach or placement of short incisions along the anatomic units is almost invisible, Figure 11.

**Figure 12:**
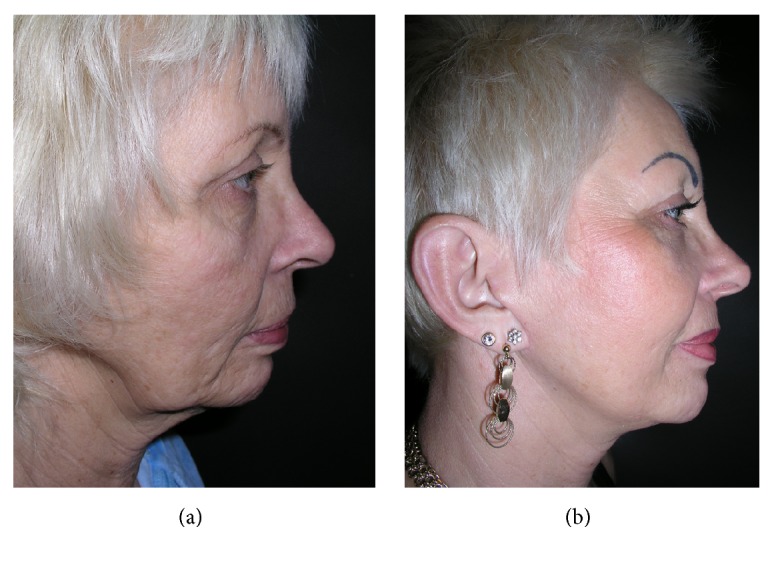
Facial rejuvenation before (a) and after (b) face-lifting.

In the majority of cases, the scars hidden in the hair-bearing skin and along its borders as well as scars placed along the Langer's lines do not appear to affect the results of surgical procedures and the favourable outcomes of classic face-liftings can be regarded as the best evidence for it, Figure 12.

Although more difficult, correction of unfavourably located linear scars does not present essential problems too, as Borghes method or various local plasties are rather efficient. Poor outcomes are very rare indeed and apart from healing problems they are related mostly to racial or individual inclination to hypertrophic scar formation.

The management of plane scars or large postexcisional wounds is much more demanding, less successful, and a priori doomed to imperfections however. As the use of local and regional flaps is rather limited, an extorted use of skin grafts or distant flaps cannot secure satisfactory outcomes and normal appearance. In large defects, the different texture, color, and patchy mosaic of reconstructed facial tegument present an ugly picture and are regarded as a real* crux chirurgorum* [14, 15].

The* timing of treatment evaluation* is also very important factor.

According to the well know saying an elapse of time should be regarded both as an ally and as a surgeon's enemy. While after many years the matured scars look better, an appearance of lifted face and neck tends to deteriorate, more so as the ptosis may be accelerateted by diseases and bad habits. Despite SMAS plasty and/or other sophisticated procedures aimed for long-lasting outcomes, 5–10 years after operation an attractive look is seemingly less evident, alas.

**Figure 13:**
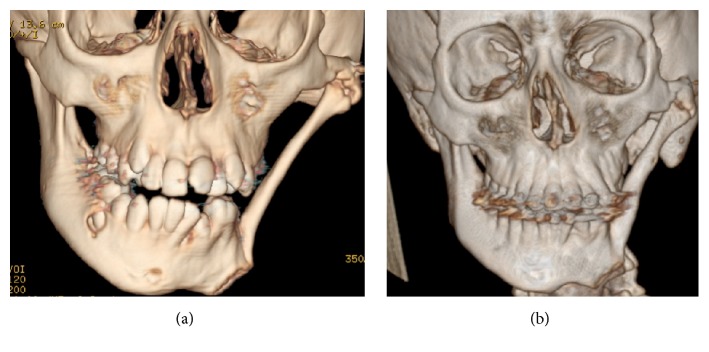
(a) Costochondral graft hypertrophy. (b) Reduction osteotomy.

In children, the treatment evaluation of HCFM varies greatly. Irrespective of adopted surgical method the best results are noted early. Later on, after distraction osteogenesis (DO) the mandibular ramus growth impairment is often observed, however, while after augmentation/reconstruction the hypertrophy of costochondral grafts can deteriorate initial results of treatment and extort reduction osteotomy [16, 17], Figure 13.

**Figure 14:**
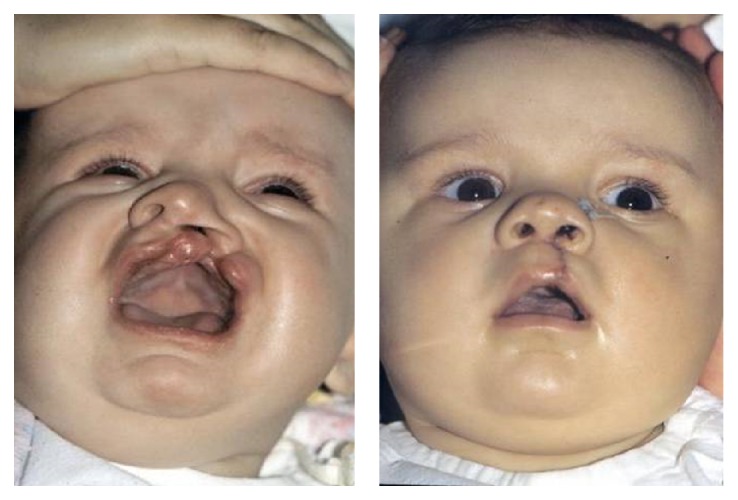
Unilateral cleft lip and nose repair.

The last factor is the* surgeon's dexterity and experience* evidenced also by proper judgement and good planning based on knowledge of numerous surgical methods. The quality of surgery cannot be overestimated. In the study of the 6th European cleft lip and palate centers conducted by Shaw et al., two variables only were found to be significant, morphology and surgical skill [18]. The surgeon's dexterity has a great practical value, because, for example, the repair outcomes of the same type of clefts happen to vary from fully acceptable to real disasters [1]. Moreover it should be remembered that the fate of these patients is largely due to the method of primary repair which should reduce the need for secondary corrections to a minimum [1], Figure 14. To a lesser degree the same applies to rhinoplasties and other cosmetic procedures.

**Figure 15:**
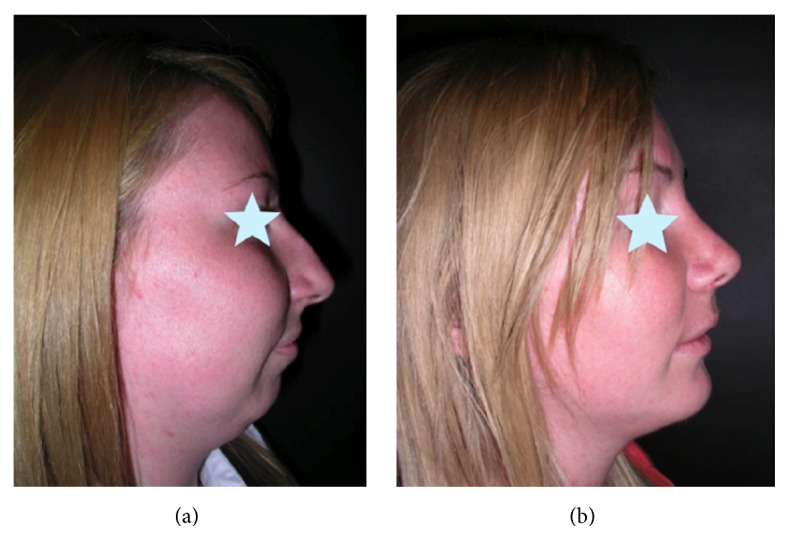
Profileplasty before (a) and after (b) simultaneous rhinoplasty and chin advancement.

The list of highly predicted perfect outcomes depending mostly on surgeon's knowledge and dexterity includes an orthognathic surgery, rhinoplasty, otoplasty, some local plasties, and a majority of rejuvenation procedures. What is important is the management of some cases demand cooperation or training in maxillofacial and other specialties, like orthodontics, prosthetics, and even neurosurgeons. It applies mostly to the most spectacular combined corrections encompassing both skeletal and soft tissue disorders, Figure 15.

Disregarding evident malpractice, in spite of favourable conditions and proper training, the* poor outcomes and complications* cannot be avoided, however. In cosmetic surgery the real failures are rare indeed, but minor imperfections are not unusual, alas [19–22].

Correction of postoperative deformities is always difficult and demanding. Apart from purely technical problems due to wasted, scarred, or displaced tissues, the dissatisfied patients' attitude restricts the planning and choice of secondary procedures because, subconsciously, still growing probability of litigation has some impact on too careful and less radical repairs.

**Figure 16:**
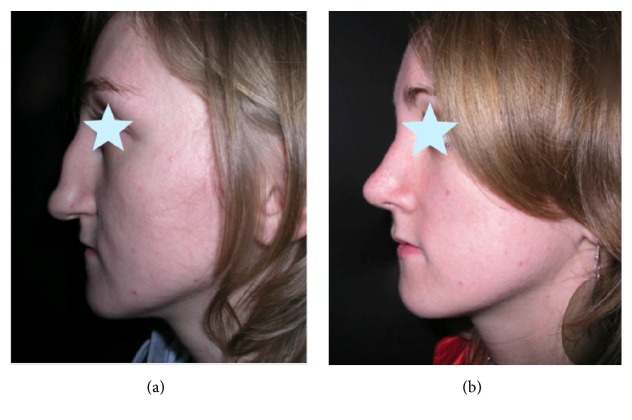
(a) Nasal deformity after unsuccessful septoplasty performed elsewhere. (b) Result of correction.

The call for secondary procedures occurs mostly after rhinoplasties, where some irregularities, impaired respiration, and asymmetry are not uncommon. In less evident cases the repair of them is relatively easy and even spectacular [19, 20], Figure 16.

The worse and extremely difficult one is correction of patients after several unsuccessful operations with deformed skeleton, overresected cartilages, and thinned, scarred skin envelope. They present a real challenge and despite repairing and/or grafting of missing tissues the fully satisfactory results are rather uncommon.

Correction of unfavourable rejuvenation procedures relates mostly to blepharoplasty, due to malposition of eyelids and poor distribution or excessive resection of fatty tissue. Despite some difficulties, the repair and achievement of attractive appearance cannot be excluded, provided the full reoperation and possibly fat grafting are performed [21, 22].

Secondary facelift operations consist also of removal of alloplastic materials, correction of too visible scars, smoothing away irregularity of subcutaneous tissue, and restoration of hair in the temporal areas. In some cases in spite of relatively short elapse of time after primary procedure, there is a need for full reoperations, which are rather reluctantly accepted by dissatisfied patients.

**Figure 17:**
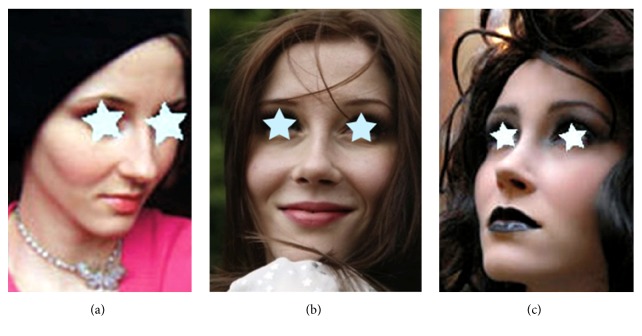
Cosmetic rhinoplasty before (a) and after (b, c) operation, without and with make-up.

The final outcomes of many operations and especially rejuvenation procedures depend also on* aesthetic medicine and beauty procedures.* Despite seemingly minor contribution, the nonsurgical and mini-invasive procedures such as Botox and filler injections or permanent make-up have a great impact on patients opinions and evaluation of treatment, Figure 17.

Some surgeons have a problem to accept it, because common evaluation of highly qualified surgery with less significant micropigmentation, new denture, or hair styling seems to be inappropriate and hardly justified. According to opinion of many colleagues it should be regarded as a* signum temporis* however and although the sharing of treatment merits seems to be rather strange, unjust, and at least debatable matter, the fillings and satisfaction of patients should be taken into consideration. On the other side, irrespective of less or more disputable contribution of “beauticians,” refined final outcomes speak always in favour of reconstructive or cosmetic surgery.

## 3. Conclusion

Despite the great potential, mitigated optimism and not excessive promises given to patients seem to be recommended and highly demanded. As a surgeons, we must not compete with mass media with unrealistic expectations, and an inflated* ego* or financial interests should not obscure the reality and honesty. Despite the proper training and modern facilities we are not almighty makers of impeccable function and appearance, since there are well known insuperable limitations and not always predicted negative factors.

As a rule, an expectation of perfect results of treatment is justified and realistic exclusively in the repair of disorders and disfigurements with nonexisting or rather limited deficit of tissue.

The need for large replacement of specific original material with substitute tissue is evidently less efficient and despite the best surgical efforts the outcomes are hardly accepted.

On the other side, we should give our patients the hope and promises within our power and an achievement of outstanding results of surgery is always highly desirable. Finally, although the common excellence can be regarded as an utopian fantasy, the efforts to be close to perfection are beneficial, stimulating, and commended.
